# “I’m not broken”: A blueprint to address stigma associated with dementia

**DOI:** 10.1002/alz70858_097741

**Published:** 2025-12-24

**Authors:** Kate Irving, Br. John‐Richard Pagan, Fernando Aguzzoli Peres, Jonathan Adrian Zegarra‐Valdivia, Walter D Dawson, Lingani Mbakile‐Mahlanza, Sherril B Gelmon

**Affiliations:** ^1^ Dublin City University, Dublin, Ireland; ^2^ Alzheimer's Disease International, Lived Expereience Panel, Woodbridge, VA, USA; ^3^ Global Brain Health Institute, Dublin, Dublin, Ireland; ^4^ Achucarro Basque Center for Neuroscience, Leioa, Spain; ^5^ Global Brain Health Institute (GBHI), University of California San Francisco, San Francisco, CA, USA; ^6^ Universidad Señor de Sipán, Chiclayo, Peru; ^7^ Global Brain Health Institute, San Francisco, CA, USA; ^8^ Oregon Health & Science University, Portland, OR, USA; ^9^ Portland State University, Portland, OR, USA; ^10^ University of Botswana, Gaborone, Botswana; ^11^ OHSU‐PSU School of Public Health, Portland, OR, USA; ^12^ GBHI Lived Experience Panel, Portland, OR, USA

## Abstract

**Background:**

The reduction of stigma is a major goal of every dementia strategy and plan published. Stigma is a concern of people living with dementia (PLWD) and their care partners. Public stigma is the perception of PLWD, which can comprise prejudice and discrimination. Self‐stigma, or internalized shame felt by the person with dementia themselves, further complicates this phenomenon. Stigma is born of fear and existential dread of loss of self that proximity to PLWD can evoke. This presentation addresses perceptions of stigma by participants in Walking the Talk for Dementia (WTD) 2024 and explores three associated sub‐themes. Collectively the findings demonstrate the evolution of destigmatisation over the week‐long event and beyond.

**Methods:**

This study combined **quantitative** and **qualitative** research approaches with the aim of providing a comprehensive understanding of the impact of WTD on participants by integrating numerical and descriptive data that addressed the issue of stigma as reported in surveys and individual reflective comments.

**Results:**

Three sub‐themes emerge from the findings: “*You no longer see them as patients*” –clinicians change their perceptions of PLWD; the “*creation of community*” – participants acknowledge their shared vulnerability; and “*I’m not broken*” – PLWD see possibilities and meaning. The themes show development of knowledge about dementia during WTD and its potential to address stigma. This often manifests as self‐knowledge born of deep reflection and connection with other participants. Crucially, the event afforded different participants to get what each needed from the event despite very diverse backgrounds and experience. The average self‐reported improvement in knowledge about the impact of dementia on individuals and society was 4.59 on a scale of 1‐5 (least to most) and 4.49 on ideas about how to advocate for PLWD.

**Conclusions:**

WTD offers a framework to address some of the fear that lies at the heart of stigma. The collective experiences and learning reported by participants offer many insights into enhanced understanding and the resulting decrease in stigmatising words or actions. People living with dementia discovered their own complicity in their stigmatisation and experienced an empowering context where they could redefine themselves in a new light free of stigma.

